# SARS-CoV-2 in the pancreas and the impaired islet function in COVID-19 patients: SARS-CoV-2 infection impairs islet function

**DOI:** 10.1080/22221751.2022.2059400

**Published:** 2022-04-18

**Authors:** Ningfei Ji, Mingshun Zhang, Liang Ren, Yunyun Wang, Bicheng Hu, Jie Xiang, Yingyun Gong, Chaojie Wu, Guoqiang Qu, Wenqiu Ding, Zhiqiang Yin, Shan Li, Zhengxia Wang, Lianzheng Zhou, Xueqin Chen, Yuan Ma, Jinhai Tang, Yun Liu, Liang Liu, Mao Huang

**Affiliations:** a Department of Respiratory and Critical Care Medicine, The First Affiliated Hospital of Nanjing Medical University, Nanjing, People’s Republic of China; b Key Laboratory of Antibody Techniques, National Health Commission, Department of Immunology, Nanjing Medical University, Nanjing, People’s Republic of China; c Department of Forensic Medicine, Tongji Medical College of Huazhong University of Science and Technology, Wuhan, People’s Republic of China; d Department of Laboratory, Wuhan No. 1 Hospital, Wuhan, People’s Republic of China; e Department of Laboratory, Wuhan Jinyintan Hospital, Wuhan, People’s Republic of China; f Diagnosis and Treatment Research Center of Wuhan Infectious Disease of Chinese Academy of Medical Sciences, Wuhan, People’s Republic of China; g Department of Endocrinology, The First Affiliated Hospital of Nanjing Medical University, Nanjing, People’s Republic of China; h Hubei Chongxin Judicial Expertise Center, Wuhan, People’s Republic of China; i Department of Dermatology, The First Affiliated Hospital of Nanjing Medical University, Nanjing, People’s Republic of China; j Department of General Surgery, The First Affiliated Hospital of Nanjing Medical University, Nanjing, People’s Republic of China; k Department of Medical Informatics, School of Biomedical Engineering and Informatics, Nanjing Medical University, Nanjing, People’s Republic of China

**Keywords:** Covid-19, sARS-CoV-2, pancreas, islet, diabetes

## Abstract

Diabetes mellitus (DM) is one of the most common underlying diseases that may aggravates COVID-19. In the present study, we explored islet function, the presence of SARS-CoV-2 and pathological changes in the pancreas of patients with COVID-19. Oral glucose tolerance tests (OGTTs) and the C-peptide release test demonstrated a decrease in glucose-stimulated C-peptide secretory capacity and an increase in HbA1c levels in patients with COVID-19. The prediabetic conditions appeared to be more significant in the severe group than in the moderate group. SARS-CoV-2 receptors (ACE2, CD147, TMPRSS2 and neuropilin-1) were expressed in pancreatic tissue. In addition to SARS-CoV-2 virus spike protein and virus RNA, coronavirus-like particles were present in the autophagolysosomes of pancreatic acinar cells of a patient with COVID-19. Furthermore, the expression and distribution of various proteins in pancreatic islets of patients with COVID-19 were altered. These data suggest that SARS-CoV-2 in the pancreas may directly or indirectly impair islet function.

## Introduction

The outbreak of coronavirus disease 2019 (COVID-19) has raised tremendous challenges. The severe acute respiratory syndrome coronavirus 2 (SARS-CoV-2) shares similarities with the SARS coronavirus [1]. SARS-CoV was reported to infect multiple organs in humans [2]. Similarly, SARS-CoV-2 was detected in the lung, pharynx, heart, liver, brain, and kidney of infected patients [3].

Diabetes mellitus (DM) is a chronic disease affecting millions of people. Concerns have been raised those diabetic patients may be at high risk for COVID-19 [4,5]. ACE2, the putative receptor for SARS-CoV-2, is rarely detected in pancreatic endocrine cells [6,7], leading to the hypothesis that SARS-CoV-2 is unlikely to directly infect pancreatic β cells *in vivo* in an ACE-2-dependent manner. In contrast, *in vitro* studies have provided evidence that human pancreatic α and β cells are susceptible to SARS-CoV-2 infection [8], implying that SARS-CoV-2 may directly target the pancreas and impair islet function. Moreover, contradictory data have shown that the SARS-CoV-2 receptors ACE2 and TMPRSS2 are expressed in pancreatic islets [9]. Although SARS-CoV-2 has been postulated to promote the occurrence of DM [10], the direct evidence linking SARS-CoV-2 with DM is still inadequate.

Hyperglycemia is commonly observed in patients with SARS [11]. Limited retrospective studies [12,13] have shown that elevation of blood glucose levels might also occur in patients with COVID-19. It is speculated that the systemic inflammatory response may contribute to the onset of DM [14,15]. SARS-CoV-2 has been detected in respiratory system[16] and kidney [3] specimens. However, the existence of SARS-CoV-2 in the pancreas and the islet function of patients with COVID-19 have not been well documented. To explore the effects of SARS-CoV-2 infection on islet function, an oral glucose tolerance test (OGTT) and C-peptide release test were performed in SARS-CoV-2-infected patients without a history of diabetes or impaired glucose tolerance. Autopsy specimens from the pancreas of patients with COVID-19 were also analyzed with immunohistochemistry (IHC), fluorescence *in situ* hybridization (FISH), and transmission electron microscopy (TEM). We found that islet function was compromised in patients with COVID-19 and that SARS-CoV-2 was present in the pancreas, suggesting that SARS-CoV-2 may directly target the pancreas and contribute to the initiation of DM.

## Materials and methods

### Study design and participants

We recruited patients with COVID-19 from March 1st to April 12th, 2020, at Wuhan No. 1. Hospital and Wuhan Jinyintan Hospital, Wuhan China. All the patients were confirmed to have SARS-CoV-2 infection with a real-time reverse transcriptase-polymerase chain reaction (RT–PCR) test. The exclusion criteria of this study included (1) a history of diabetes, prediabetes, or taking medicine to control blood sugar before COVID-19; (2) cancer; (3) pancreatic diseases (acute pancreatitis, chronic pancreatitis or pancreatic injury); (4) autoimmune disease; (5) immunodeficiency; (6) glucocorticoid treatment within 6 months before admission; and (7) pregnancy or breastfeeding. None of the patients received glucocorticoid treatment during hospitalization. All patients were provided with enough carbohydrate intake for a balanced diet, and none were prescribed parenteral nutrition or nasal feeding.

### Study approval

The study was approved by the ethics committee of the First Affiliated Hospital of Nanjing Medical University, Wuhan No. 1 Hospital, Wuhan Jinyintan Hospital and Tongji Medical College of Huazhong University of Science and Technology (2020-SR-134, KY-2020-15.01 and KY-2020-52.01). Written informed consent was obtained from all patients.

### Clinical procedures

Epidemiological, demographic, and baseline characteristics and laboratory results were obtained from patients’ medical records. Inflammatory factors, including C-reactive protein (CRP) and IL-6, were routinely measured. The 75-g OGTT was performed. Briefly, after at least 8 h of fasting, the patients donated blood to measure fasting plasma glucose and glycosylated hemoglobin A1c (HbA1c) levels. Water-free glucose powder (75 g) was dissolved in 200 ml of drinking water and was consumed in 5 min. The timer was set as 0 min when the patient drank the first sip. Then, blood samples were collected at 30-, 60-, 120-, and 180-min post-glucose consumption. Plasma glucose and C-peptide were measured to determine glucose tolerance and the secretory capacity of pancreatic islets. According to the glucose metabolism levels announced by the World Health Organization (WHO) in 1999 [17], subjects with fasting blood glucose (FBG) < 6.1 mmol/L and 2-h blood glucose (2hBG) < 7.8 mmol/L were grouped into normal glucose tolerance; those with FBG ≥ 7.0 mmol/L and 2hBG ≥11.1 mmol/L were in the diabetes group; and those with blood levels not fitting in the above two groups were in the prediabetes group.

### Opal immunofluorescence staining in pancreatic samples

Autopsy samples were collected from four patients with COVID-10, and opal multiplex immunofluorescence staining was processed as described previously [18,19]. Briefly, formalin-fixed paraffin-embedded (FFPE) pancreatic tissue samples were cut into 3-μm-thick serial sections and further stained for simultaneous detection and quantitation of ACE2 (Ab108252, 1:200, Opal 570 channel, pseudo-yellow), NKX6.1a (CST#54551S, 1:100, Opal 520 channel, pseudo-green), CD147 (Ab10830, 1:500, Opal 690 channel, pseudo-magenta), neuropilin-1 (ab81321, 1:100, Opal 620 channel, pseudo-red), TMPRSS2 (Abcolonal, A9126, 1:400, Opal 780 channel, pseudo-white) and nucleus (DAPI, pseudo-blue) by using an Opal Polaris 7 Color Automation IHC Detection Kit (Akoya Biosciences, Menlo Park, CA). The slides were observed and imaged by a Vectra Polaris automated quantitative pathology imaging system. The images were sequentially spectrally unmixed by Akoya phenoptics inForm software (inform 2.4.8).

### Immunohistochemistry (IHC) of pancreatic samples

Autopsy samples were collected from four patients with COVID-19 and processed as described previously [16]. Briefly, FFPE pancreatic tissue samples were cut into 3-μm-thick serial sections. Following antigen retrieval in EDTA (pH: 9.0), the sections were incubated overnight at 4 °C with primary antibodies against SARS-CoV-2 spike (1:200, GTX632604, Genetex), ACE2 (1:200, GB11267, Servicebio), CD147 (1:200, GB11390-1, Servicebio), PDX1 (1:200, 20989-1-ap, Proteintech), PPAR (1:200, BS-4590R, BIOSS), CD36 (1:200, 18836-1-ap, Proteintech), GLUT2 (1:200, bs-051r, BIOSS), IRS1 (1:200, AF6273, Affinity), or IRS2 (1:200, bs-0173r, BIOSS). After extensive washing, the sections were further stained with the corresponding secondary antibodies and visualized using the Dako REAL™ EnVision™ Detection System. Dilutants without primary antibodies were used as negative controls.

### Fluorescence in situ hybridization (FISH) of pancreatic samples

Pancreatic samples obtained from autopsy were fixed with 4% PFA in diethyl pyrocarbonate (DEPC) for 8 h. After dehydration, the fixed tissue sample was cut into 3-μm-thick serial sections. Following digestion in proteinase K (20 μg/ml) at 37 °C for 20 min, the sections were incubated with 6 ng/μl SARS-CoV-2 probe (5’-CY3-CCGUC UGCGG UAUGU GGAAA GGUUA UGG-3’) at 37 °C overnight. After washing, the FISH preparations were counterstained with DAPI and observed by confocal microscopy with appropriate fluorescence filter sets (Nikon, Japan).

### Transmission electron microscopy (TEM)

TEM was utilized to detect changes in the ultrastructure and structure of the SARS-CoV-2 virus particles [16]. Briefly, fresh pancreatic tissues (approximately 1 mm×1 mm×1 mm in size) were fixed in 3% buffered glutaraldehyde in 0.1 M phosphoric buffer (pH: 7.4) for 2∼4 h and 1% (w/v) osmic acid for 2 h, dehydrated with gradient alcohol, and embedded in Epon 812 (SPI, PA, USA). After polymerization of the resin at 60 °C for 48 h, ultrathin sections were cut at 70-nm thickness with an ultramicrotome using a diamond knife, stained with 5% uranyl acetate and lead citrate, and observed under a Hitachi HT7700 transmission electron microscope.

### Statistical analysis

All data, as appropriate, are expressed as the mean ± standard deviation or percentages. The mean and percentages between groups were compared using ANOVA and the chi-square test in SPSS (version 22.0). The calculation formulas for evaluating insulin resistance are listed as follows: C-peptide index = fasting C-peptide (mmol/L)/fasting blood glucose (mmol/L)*100[20]; 20/(fasting C-peptide (nmol/L)    fasting glucose (mmol/L))[21].

## Results

### Islet function was compromised in patients with covid-19

A total of 42 patients with COVID-19, 21 males and 21 females, were recruited from two medical centers in Wuhan for the study (Table 1). The patients ranged from 23 to 93 years old. Of the 42 patients, the classification of COVID-19 disease severity from the Clinical Criteria of the WHO [22] was as follows: 1 was mild, 22 were moderate, 18 were severe, and 1 was critical. We divided the patients into two groups based on their disease severity: the 23 mild and moderate patients were named the moderate group, while the 19 severe and critical patients were named the severe group (Table 2). To explore whether SARS-CoV-2 infection impaired systemic glucose tolerance and islet function, we performed OGTT tests and C-peptide secretion tests on patients with COVID-19. The patients in the severe group were older than the patients in the moderate group (65.79 ± 14.73 *vs.* 52.78 ± 15.37, *p *=  0.008), reflecting that age was a risk factor for the disease severity of COVID-19. A higher body mass index (BMI) is a common risk factor for type 2 diabetes mellitus (T2DM) [23], and it was comparable between the two groups (21.70 ± 2.67 *vs.* 23.22 ± 3.84, *p *= 0.202).

**Table 1.** Clinical characteristics and laboratory findings in patients with COVID-19.

	Wuhan No.1	Jinyintan	Total	Reference range
Gender(M/F)	11/6	10/15	21/21	
Age (years old)	68.6 ± 12.7	52.28 ± 15.57	58.66 ± 16.48	
Underlying diseases				
hypertension	0	2	2	
hyperuricemia	0	1	1	
No potential comorbidities	17	22	39	
Disease Severity				
mild disease	0	1	1	
moderate disease	1	21	22	
severe disease	15	3	18	
critical disease	1	0	1	
Hospitalization days^1^				
<30 days	2	0	2	
30∼60 days	14	12	27	
>60 days	1	13	13	
Blood routine				
Leukocyte (×10^9^/L)	7.01 ± 0.58	5.49 ± 0.32	6.13 ± 0.32	3.50-9.50
Neutrophils (×10^9^/L)	4.43 ± 0.63	3.05 ± 0.23	3.63 ± 0.31	1.80-6.30
Lymphocytes (×10^9^/L)	1.58 ± 0.21	1.85 ± 0.12	1.74 ± 0.11	1.10-3.20
Blood chemistry				
TBIL (μmol/L)	ND	10.9 ± 3.5	NA	1.71-21
Albumin (g/L)	36.06 ± 8.11	39.8 ± 3.71	38.46 ± 5.87	40–55
Globulin (g/L)	28.00 ± 6.16	27.08 ± 2.55	27.44 ± 4.28	20–40
ALT (IU/L)	28.7 ± 3.6	29.4 ± 4.3	29.1 ± 2.9	7–45
AST (IU/L)	24.3 ± 2.0	27.3 ± 2.5	26.1 ± 1.7	13–35
Creatinine (μmol/L)	70.4 ± 5.6	60.3 ± 4.1	64.5 ± 3.4	44–97
BUN (mmol/L)	5.7 ± 0.9	4.5 ± 0.2	5.0 ± 0.4	1.8-7.3
Inflammation parameters				
CRP (mg/L)	17.38 ± 24.92	1.58 ± 2.03	7.75 ± 17.21	≤5
IL-6 (pg/ml)	9.50 ± 10.67	6.74 ± 2.53	7.66 ± 6.46	≤5
Treatment				
antiviral^2^	17	0		
antibiotics^3^	16	1		

^1^ The infection history for patients in Jingyintan Hospital cohort study was over 30 days.

^2^ Patients were treated with Arbidol, Oseltamivir, Kaletra, or Hydroxychloroquine.

^3^ Patients were treated with Meropenem, Moxifloxacin, Tegafycline or Azithromycin.

Abbreviation: NLR, neutrophil-to-lymphocyte ratio; TBIL, total bilirubin; ALT, alanine aminotransferase; AST, aspartate aminotransferase; BUN, blood urea nitrogen; CRP, C-reactive protein; IL-6, interleukin-6; ND, not done; NA, not available

**Table 2.** Results of the oral glucose tolerance test in patients with COVID-19.

	Moderate group (n = 23)	Severe group (n = 19)	*P* value	Reference range
Age (years)	52.78 ± 15.37	65.79 ± 14.73	0.008	
BMI (kg/m^2^)	23.22 ± 3.84	21.70 ± 2.67	0.202	
Glucose (mmol/L)				
0 min	4.95 ± 0.50	5.39 ± 1.02	0.238	3.9-6.1
30 min	8.80 ± 2.09	9.40 ± 2.09	0.433	6.1-9.4
60 min	9.09 ± 2.22	9.92 ± 2.92	0.463	6.7-9.4
120 min	7.63 ± 1.96	8.97 ± 4.38	0.595	3.9-7.8
180 min	5.68 ± 1.70	7.82 ± 5.41	0.390	3.9-6.1
C-peptide (ng/ml)				
0 min	1.97 ± 0.95	2.37 ± 1.01	0.060	0.30-0.61
30 min	7.76 ± 4.52	6.45 ± 3.07	0.536	1.5-6.1
60 min	8.65 ± 3.34	9.30 ± 5.69	0.791	1.5-6.1
120 min	9.71 ± 3.77	9.75 ± 6.38	0.640	
180 min	7.72 ± 3.83	7.32 ± 4.71	0.471	0.30-0.61
HbA1c (%)	5.15 ± 0.37	6.12 ± 1.60	0.001	4∼6%
Glu_AUC	1375.24 ± 272.38	1582.18 ± 588.38	0.587	
Cp_AUC	1543.73 ± 547.45	1451.97 ± 822.95	0.411	
CPI	1.32 E-4 ± 6.98 E-5	1.51 E-4 ± 7.09 E-5	0.153	
20/(FCp*FBG)	7.45 ± 2.94	6.19 ± 4.07	0.063	
Cpmax/FCp	6.14 ± 1.79	4.78 ± 1.63	0.014	

Abbreviations: HbA1c, hemoglobin A1c; Glu_AUC, area under the curve of glucose in the OGTT; Cp_AUC, area under the curve of C-peptide in the OGTT; Cp, C-peptide; CPI, C-peptide index; FCp, fasting C-peptide; FBG, fasting blood glucose; Cpmax, the maximum value of C-peptide during the OGTT.

**Figure 1.**
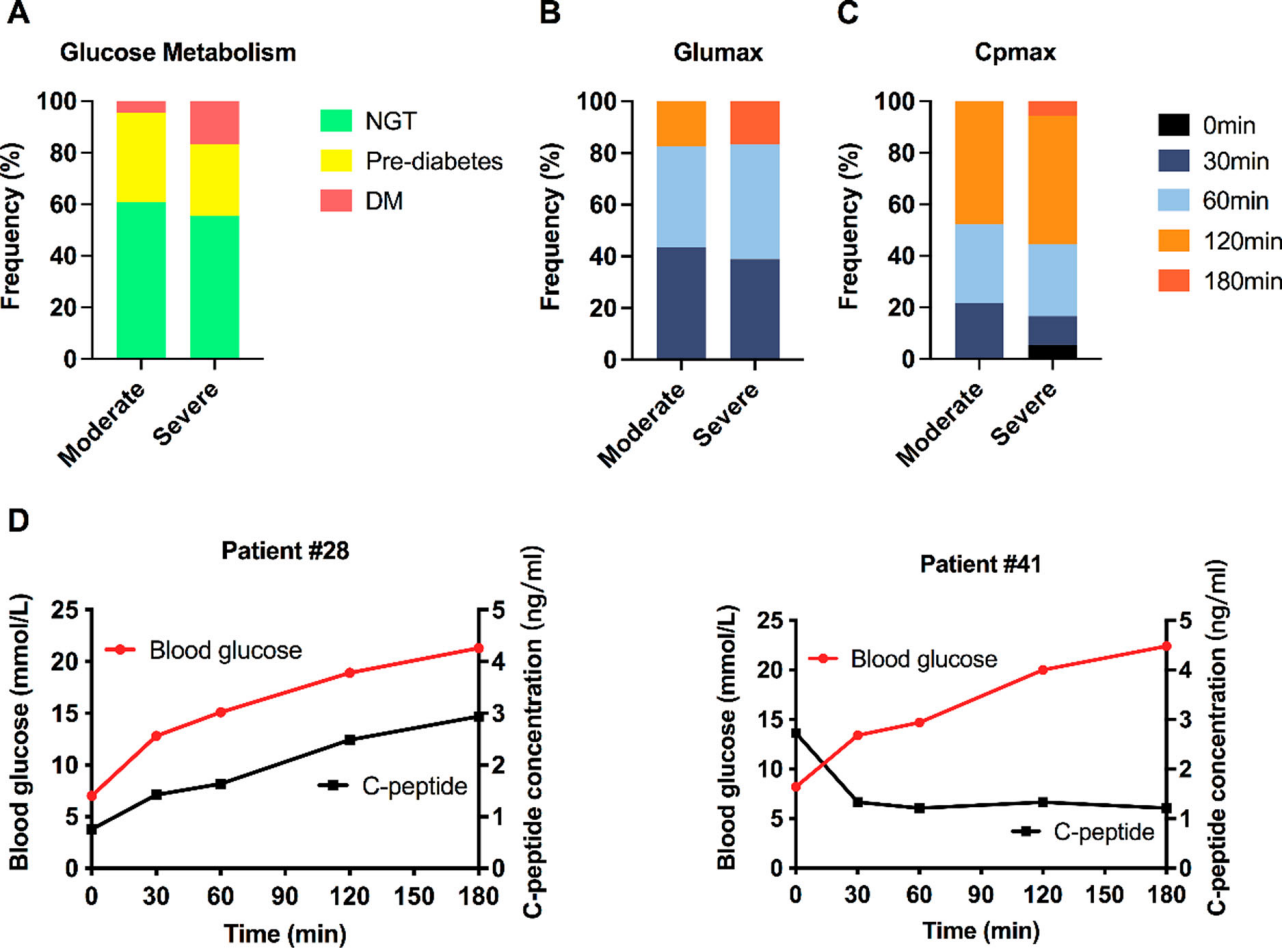
Distribution of normal glucose tolerance, prediabetes and diabetes after COVID-19. (A) Distribution of patients with COVID-19 into normal glucose tolerance (NGT), prediabetes or diabetes mellitus (DM) groups. During the OGTT, the peak time distributions of blood glucose (B) and C-peptide (C) in the mild-moderate group and the severe-critical group were analyzed. (D) OGTT results of 2 representative patients with COVID-19 and new-onset diabetes. In patient #29, C-peptide (black curve, right axis, ng/ml) was released in response to the elevation of blood glucose levels (red curve, left axis, mmol/L). In patient #42, the blood glucose level was increased, but the C-peptide level was very low throughout the entire test.

The HbA1c level was significantly higher in the severe group than in the moderate group (6.1 ± 1.6 *vs.* 5.1 ± 0.4, *p *= 0.001). In the OGTT test, the average fasting blood glucose (FBG) concentration in the severe group was slightly higher than that in the moderate group (5.4 ± 1.0 *vs.* 4.9 ± 0.5, *p *= 0.238), but was not statistically significant. Similar results were found for the blood glucose concentrations 30-, 60-, 120-, and 180-min post-glucose consumption. Accordingly, 14 patients (60.9%) in the moderate group had normal glucose tolerance (NGT), 9 showed characteristics of prediabetes, and 1 was newly diagnosed with DM. In contrast, 3 patients were diagnosed with new-onset diabetes, 5 patients suffered from prediabetes, and 10 patients in the severe group had NGT (Figure 1A). The chi-square test did not show a significant difference in the composition ratio between the mild-moderate and severe-critical groups according to glucose tolerance levels. Collectively, 18 of the 42 patients with COVID-19 (18/42 = 42.86%) developed prediabetes or diabetes. The peak time of blood glucose or C-peptide concentration indicated that the patients in the severe group seemed to respond slower than those in the moderate group (Figure 1B and C), although the difference did not reach statistical significance. To our surprise, the level of C-peptide was very low in 1 patient (patient #41) diagnosed with new-onset diabetes. The second patient (patient #28) with newly diagnosed diabetes had impaired insulin secretion and failed to lower glucose levels to a normal range (Figure 1D), indicating an insulin resistance. These data suggest that SARS-CoV-2 might promote the development of diabetes.

Similar to blood glucose, C-peptide is a surrogate marker for pancreatic islet function, reflecting insulin secretion. As shown in Table 2, the average baseline level of C-peptide (0 min) was slightly higher in the severe group than in the moderate group, indicating a higher fasting insulin concentration, an indicator of systemic insulin resistance. Insulin is a rapid-acting hormone with a short half-life of approximately 4–6 min. Moreover, C-peptide measurements will be significantly influenced by sample hemolysis, which means that the repeatability and accuracy may vary in different labs. Alternatively, C-peptide is simultaneously released with insulin, remaining stable for 24 hours in separated serum and plasma samples, thus becoming a more reliable marker for indicating insulin secretion capacity. Two indices based on serum C-peptide contents were applied in the current study, including the C-peptide index (CPI) and the 20/(fasting C-peptide*fasting blood glucose) ratio (20/FCp*FBP), which are indicators for beta-cell function and insulin sensitivity, respectively. Moreover, it has even been proven that 20/FCp*FBP performs better than the widely used index HOMA-IR, especially in those with mild insulin resistance [21]. As shown in Table 2, 20/FCP*FBP showed a reduction tendency in the severe group (7.45 ± 2.94 *vs.* 6.19 ± 4.07, *p *= 0.063), while CPI was comparable between the two groups. Moreover, the maximum levels of C-peptide during the OGTT/FCp (Cpmax/FCp) was significantly decreased in patients with severe COVID-19 (6.14 ± 1.79 *vs.* 4.78 ± 1.63, *p *= 0.014). Collectively, these data suggested that SARS-CoV-2 infection may impair islet functions, especially in patients with severe COVID-19.

### SARS-CoV-2 virus was detected in the pancreas of patients with covid-19

**Figure 2.**
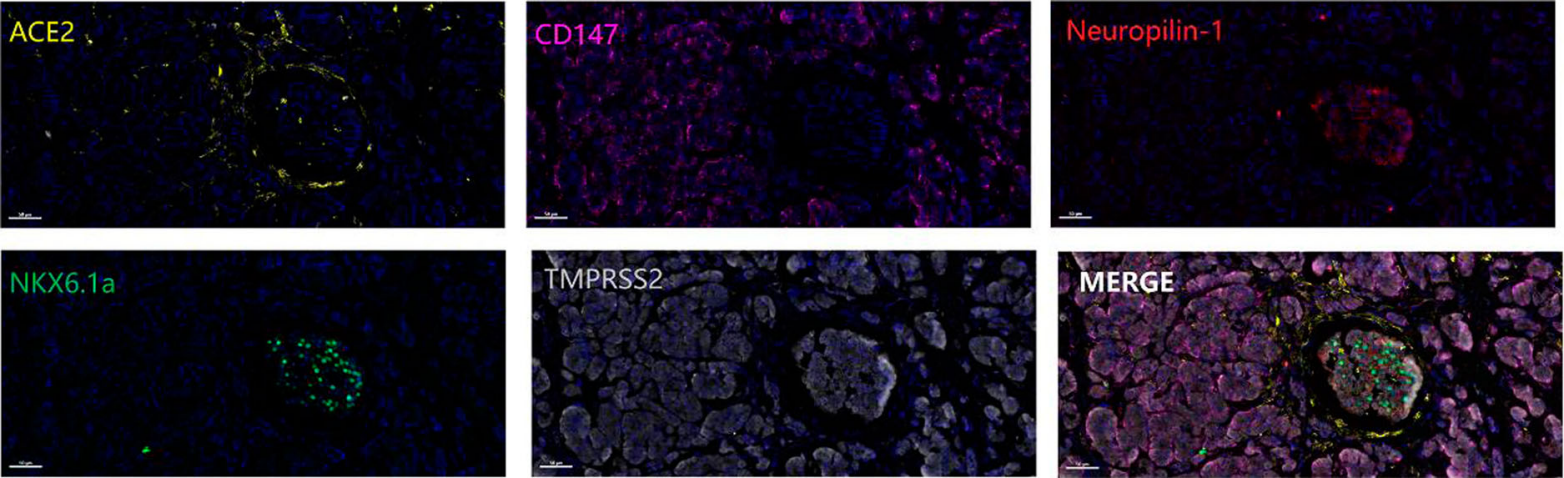
Opal immunofluorescence staining of SARS-CoV-2 receptors in pancreatic samples. The SARS-CoV-2 receptors ACE2 (pseudo-yellow), CD147 (pseudo-magenta), neuropilin-1 (pseudo-red), and TMPRSS2 (pseudo-white) and the pancreatic β cell maker NKX6.1a (pseudo-green) were detected in the pancreatic samples. Scale bar, 50 μm.

ACE2 [24], CD147 [25], neuropilin-1 [26] and TMPRSS2 [27] are receptors for SARS-CoV-2. To explore the possibility that SARS-CoV-2 may infect pancreatic cells directly, we first examined the expression of SARS-CoV-2 receptors in pancreatic autopsy samples. As shown in Figure 2, ACE2, CD147 and TMPRSS2 were widely expressed on the cell membrane in the pancreas. NKX6.1a, as a critical regulator of pancreatic β cells [28], was clearly detected only in islets. In contrast, neuropilin-1 was weakly expressed in islets. The expression of SARS-CoV-2 receptors in pancreatic tissues and islets suggested that islets may be susceptible to SARS-CoV-2 infection.

**Figure 3.**
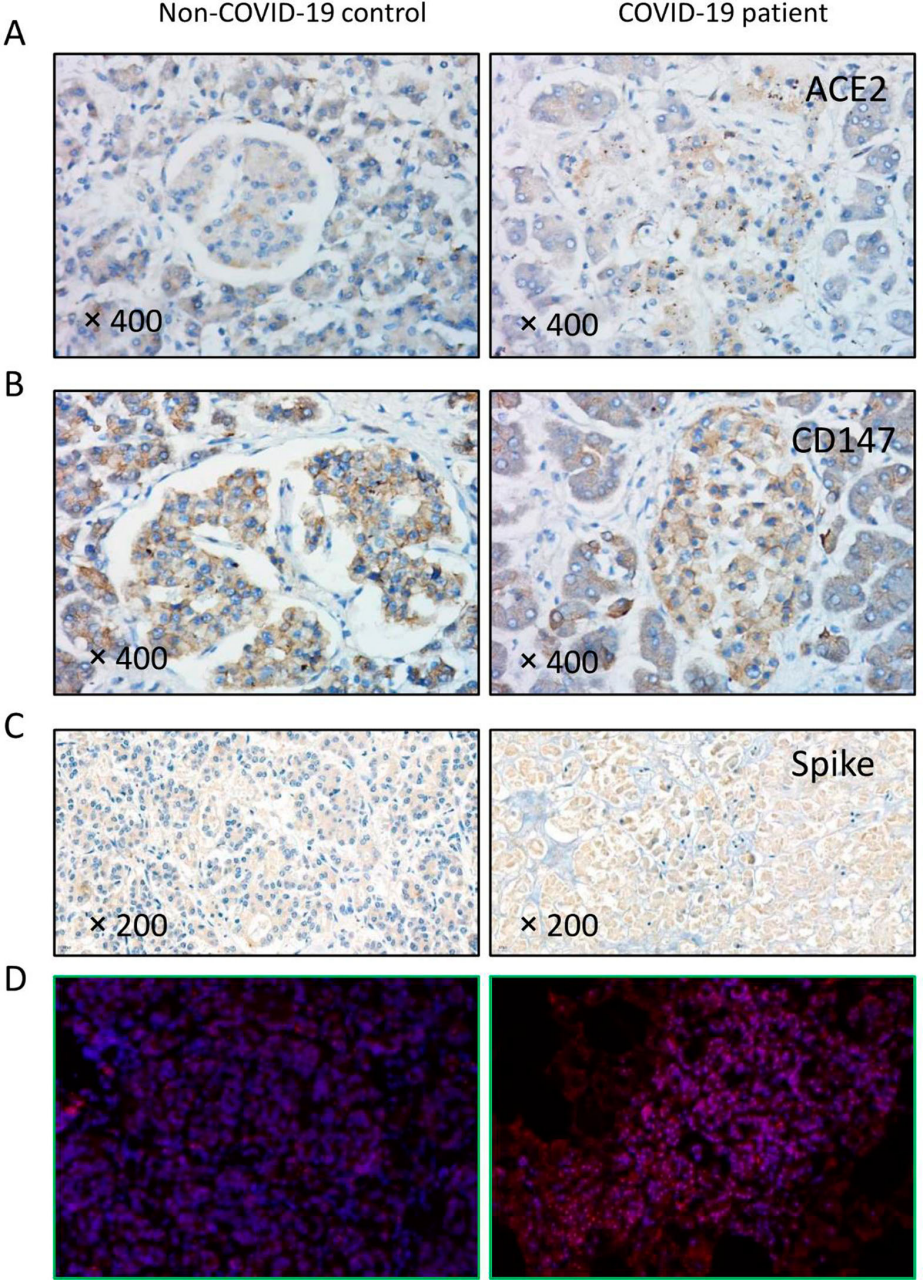
SARS-CoV-2 in the pancreas of COVID-19 patients. (A-B) SARS-CoV-2 receptors ACE2 and CD147 were diffusely expressed in the pancreas. In the non-COVID-19 control patient, ACE2 expression was higher in the exocrine gland, and CD147 was evenly expressed. In patients with COVID-19, ACE2 and CD147 expression was much higher in the endocrine gland. (C) SARS-CoV-2 spike antibody staining showed diffuse positive signals (brown) in the sample from the patient with COVID-19 but not in the non-COVID-19 control. (D) The SARS-CoV-2 nucleic acid probe showed positive staining (red particles) in the cytoplasm of pancreatic cells of a patient with COVID-19 but not in that of the non-COVID-19 control.

To further compare the difference between non-COVID-19 and COVID-19 autopsy samples, we performed immunohistochemical analysis in patients with COVID-19, and the expression of ACE2 and CD147 seemed to be higher in the endocrine gland (islet). SARS-CoV-2 spike proteins were widely distributed in the parenchyma of the pancreas of patients with COVID-19 but not in the non-COVID-19 control samples. To further confirm this observation, we performed fluorescence *in situ* hybridization (FISH) analyses using a nucleic acid probe specific for SARS-CoV-2 RNA. We detected positive fluorescent signals in the cytoplasm of pancreatic cells of the patients with COVID-19 but not in the non-COVID-19 controls (Figure 3).

**Figure 4.**
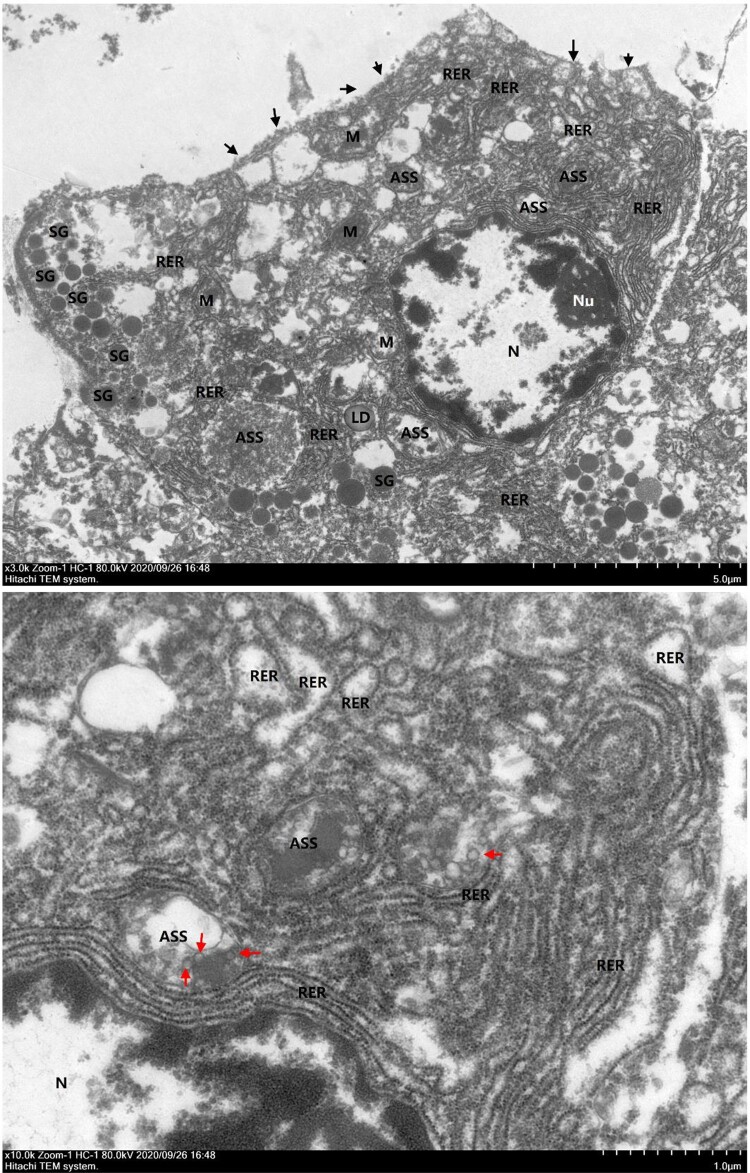
Transmission electron microscopic examination of pancreatic samples. Black solid triangles (▴) indicate cell membrane rupture. Red solid triangles (▴) indicate virus-like particles in the autophagolysosome (ASS). M, mitochondria; N, nucleus; Nu, nucleoli; RER, rough endoplasmic reticulum; SG, secretory granules.

With the aid of transmission electron microscopy, we directly observed cell damage and virus-like particles in the pancreas (Figure 4). The overall structure of acinar epithelial cells was significantly edema and disintegrated, a large area of cell membrane was damaged and dissolved, intracytoplasmic organelles were swollen, vacuolated and transformed nuclei were irregularly shaped with local pits, euchromatin dissolved heterochromatin edge set, nucleoli were large, and the nuclear membrane was clear. The mitochondria were obviously swollen, most of them were enlarged, and the stromal lysis ridge disappeared. The coarse endoplasmic reticulum was abundant, and some parts of the ER were obviously expanded and degranulated. There were fewer secretory granules, the uniform density of cell connection disappeared, and the cell gap was significantly widened. Moreover, a small number of circular structures could be seen in the autophagolysosomes, which were suspected to be virus-like particles. Collectively, these data suggest that SARS-CoV-2 may directly infect islet cells.

### Altered expression patterns of islet function-related molecules in the pancreas of patients with covid-19

**Figure 5.**
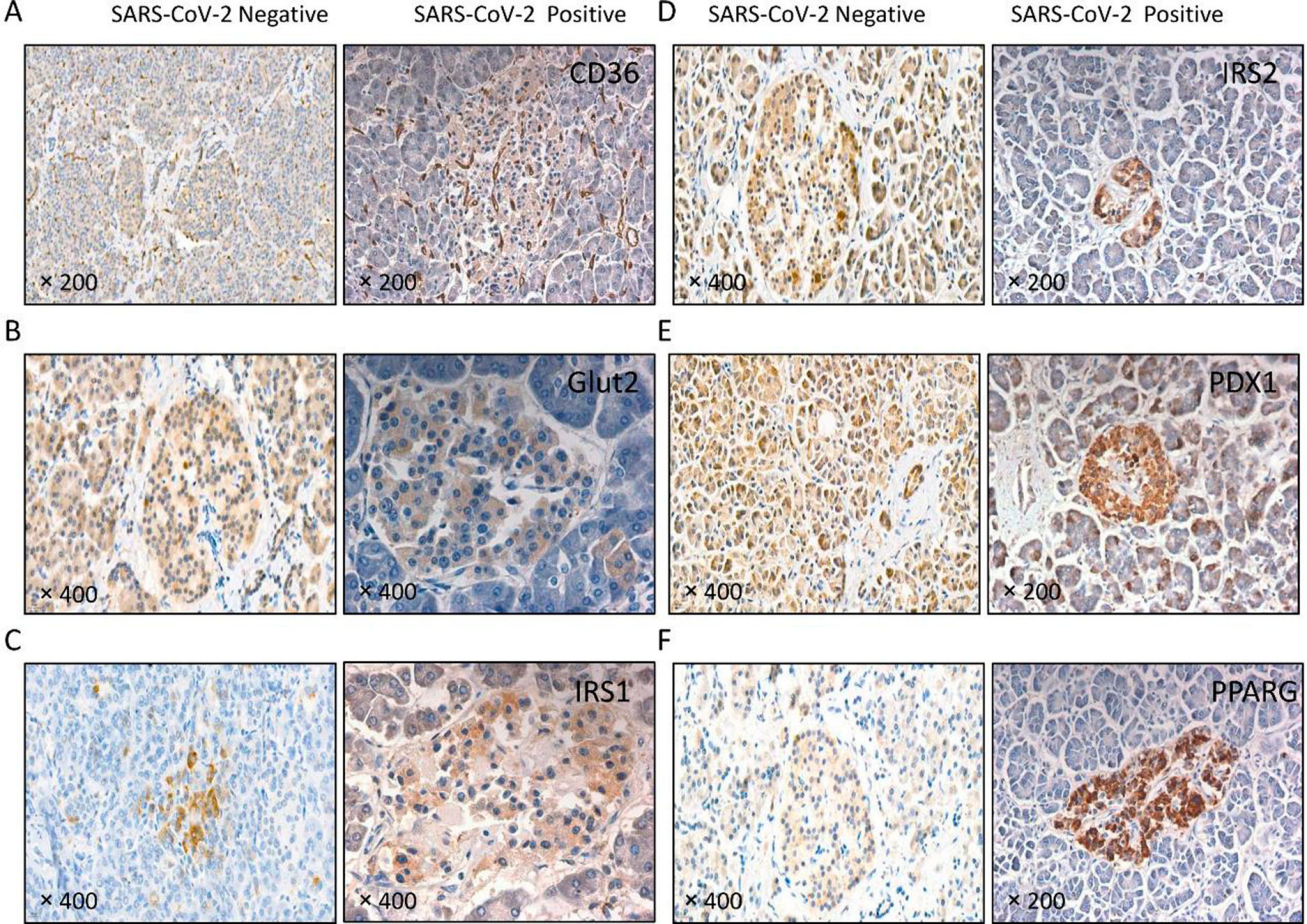
The expression of islet function-related molecules was altered in patients with COVID-19. Immunochemical staining using antibodies against (A) CD36, (B) GLUT2, (C) IRS1, (D) IRS2, (E) PDX1 and (F) PPARG. In the patient with COVID-19 but without SARS-CoV-2 virus in the pancreas (SARS-CoV-2 negative), CD36, GLUT2, IRS2 and PDX1 were widely distributed in the pancreatic serous acinus, the duct (exocrine gland) and the islet (endocrine gland); PPARG was mainly in the islet; and IRS1 was barely detected. In the patient with COVID-19 accompanied by SARS-CoV-2 virus in the pancreas (SARS-CoV-2 positive), CD36, GLUT2, IRS1, IRS2, PDX1 and PPARG were mainly detected in the islets. CD36 was also present in the small vasculature of pancreatic interstitial tissue.

Many molecules are involved in islet function and the pathogenesis of diabetes, including CD36, glucose transporters (GLUT) 2, insulin receptor substrate (IRS) 1, IRS2, pancreatic and duodenal homeobox 1 (PDX1), and peroxisome proliferator activated receptor gamma (PPARG) [29–33]. In the COVID-19 patient without SARS-CoV-2 virus expression in the pancreas (SARS-CoV-2 negative), CD36, GLUT2, IRS2, and PDX1 were widely distributed in the pancreatic, serous acini, duct (exocrine gland) and islet (endocrine gland). PPARG was mainly expressed in the islet, while IRS1 was barely detected. In the patient with SARS-CoV-2 virus expression in the pancreas (SARS-CoV-2 positive), CD36, GLUT2, IRS1, IRS2, PDX1, and PPARG were mainly detected in the islet. CD36, IRS1, and PDX1 were also weakly expressed in the exocrine gland. Of note, CD36 was abnormally expressed in the small vasculature of pancreatic interstitial tissue of SARS-CoV-2-infected patients accompanied by SARS-CoV-2 virus in the pancreas. In summary, the expression patterns of CD36, GLUT2, IRS1, IRS2, PDX1, and PPARG in the pancreas were altered upon SARS-CoV-2 infection (Figure 5), implying that islet function may be compromised in patients with COVID-19.

## Discussion

The mortality rate of COVID-19 is estimated to be 1-4%, which is significantly lower than that of SARS (∼9%) [34] and MERS (∼35%) [35]. However, COVID-19 may become more fatal for patients with diabetes and other underlying diseases, e.g. hypertension and chronic obstructive pulmonary disease (COPD). The COVID-19 case fatality rate accompanied by diabetes was 7% [36]. The increased mortality rate may be caused by the prevalence of diabetes in elderly patients or the interactions between SARS-CoV-2 and diabetes. In this pilot study, SARS-CoV-2 was found in the pancreas, and islet function was impaired in patients with COVID-19, suggesting that SARS-CoV-2 infection may promote the occurrence of DM.

SARS-CoV-2 virus particles have been found in the lungs [16], kidney [37,38], brain [39], and feces [40]. A cohort autopsy study identified SARS-CoV-2 in multiple organs, including the pancreas [41]. In the present study, we detected SARS-CoV-2 receptors (ACE2 and CD147), spike protein, viral nucleic acids, and intact coronavirus-like particles in the pancreas of patients with COVID-19. Our observation strengthened the previous finding that pancreatic cells in organoid culture were permissive to SARS-CoV-2 infection [8]. Moreover, SARS-CoV-2 virus particles were mainly present in the autophagolysosomes of infected acinar cells, indicating that autophagy may be involved in SARS-CoV-2 infection of the pancreas. Indeed, SARS-CoV-2 infection may cause the accumulation of autophagosomes [42]. The mechanisms and potential roles of autophagy in SARS-CoV-2 infection of the pancreas need further investigation.

As a scavenger receptor for free fatty acids, CD36 is widely expressed on β cells and α cells in pancreatic islets and contributes to insulin resistance and diabetes [29]. GLUT2 is required for glucose-stimulated insulin secretion in pancreatic islet β cells [30, 43]. IRS1 and IRS2 mediate the growth and function of pancreatic islet β cells [31]. IRS2 is especially crucial in insulin sensitivity and is responsible for initiating the progression of T2DM [44]. Similarly, PDX1 regulates pancreatic development and pancreatic islet β cell function [45]. PPARG plays diverse roles in the pathogenesis of diabetes, regulating adipogenesis, lipid metabolism, insulin sensitivity, and inflammation [33]. We found that SARS-CoV-2 infection in the pancreas enriched the expression of CD36, GLUT2, IRS2, and PDX1 in the pancreatic islets, which were widely distributed in the SARS-CoV-2-negative pancreas. IRS1 was found to be expressed in the pancreatic islets of the SARS-CoV-2-positive pancreas but was barely detectable in the SARS-CoV-2-negative pancreas. Moreover, CD36 was abnormally expressed in the small vasculature of pancreatic interstitial tissue of SARS-CoV-2-infected patients accompanied by SARS-CoV-2 expression in the pancreas, which might damage endothelial cells. All of these molecules are closely associated with pancreatic islet β cell function, implying that SARS-CoV-2 infection may directly or indirectly alter pancreatic islet function.

To evaluate the islet function in patients with COVID-19, we performed OGTT and C-peptide release tests. C-peptide is usually released simultaneously with the secretion of insulin. C-peptide levels are normally rather low in patients with type 1 diabetes mellitus (T1DM), and its secretion is slow in reaction to acute glucose stimulation in patients with T2DM. Patient #42 in our study had low C-peptide levels not only at baseline but also after OGTT, which were similar to the phenotypes of subjects with T1DM [46]. On the other hand, patient #29 developed insulin resistance, which is the hallmark of T2DM. However, we were unable to subtype these two patients into either T1DM or T2DM without other clinical indices, such as islet autoantibodies, and responses to insulin treatment. HbA1c provides a reliable measure of chronic glycemia in patients from the previous 2–3 months of treatment and is largely influenced by glucose levels during the last month of treatment. In our study, most of the patients were hospitalized for approximately 1 month. Thus, HbA1c was regarded as a good indicator of overall glucose metabolism during COVID-19 infection. The level of HbA1c was increased, especially in patients with severe or critical COVID-19, and was in line with the observation that the blood glucose level was higher in patients with severe or critical COVID-19. Although HbA1c is routinely detected in diabetic patients [47], some other conditions may also lead to elevated HbA1c in the absence of long-term increase in blood glucose levels [48]. In line with our observations, a case report described the occurrence of DM following SARS-CoV-2 infection [49]. Emerging evidence supports that SARS-CoV-2 may induce hyperglycemia [50] and new-onset insulin resistance [51]. More studies are needed to better understand the roles and mechanisms of SARS-CoV-2 infection and COVID-19 in the initiation and progression of diabetes.

Our study has some limitations. 1) In the Wuhan outbreak of SARS-CoV-2, most hospitals received only COVID-19 patients. Therefore, COVID-19 patients alone were recruited in the study, and non-COVID-19 controls were lacking in the OGTT. 2) Absence of evidence is not evidence of absence. The islet conditions in recovered COVID-19 patients were clear, which may limit the effects of SARS-CoV-2 infection on the pancreas. 3) We followed up with 6 patients for up to 2 years. Considering that abnormal glycemia reverts to normal in recovered COVID-19 patients [52], it is unexpected that five survivors still had impaired glucose tolerance. More patients should be followed up for a longer time. 4) Due to the limited number of autopsies, we could not convincingly correlate virus proteins and inflammation markers in pancreatic tissue with disease severity and prognosis.

In this pilot study, OGTT and C-peptide release tests demonstrated that pancreatic islet function was impaired in patients with COVID-19, especially patients with severe or critical disease. SARS-CoV-2 virus particles were detected in the pancreas of patients with COVID-19, accompanied by altered expression of CD36 and other molecules potentially contributing to the pathogenesis of diabetes. Collectively, our data suggested that SARS-CoV-2 was present in the pancreas and that islet function was compromised in patients with COVID-19. The controversial relationship between the occurrence of diabetes in patients with COVID-19 and SARS-CoV-2 infection [53] warrants further study.

## Data Availability

The data that support the findings of this study are available from the corresponding author upon reasonable request.
